# Comprehensive characterization of coding and non-coding single nucleotide polymorphisms of the Myoneurin (MYNN) gene using molecular dynamics simulation and docking approaches

**DOI:** 10.1371/journal.pone.0296361

**Published:** 2024-01-02

**Authors:** Sadia Islam Mou, Tamanna Sultana, Dipankor Chatterjee, Md. Omar Faruk, Md. Ismail Hosen

**Affiliations:** Department of Biochemistry and Molecular Biology, University of Dhaka, Dhaka, Bangladesh; University of Michigan, UNITED STATES

## Abstract

Genome-wide association studies (GWAS) identified a coding single nucleotide polymorphism, MYNN rs10936599, at chromosome 3q. MYNN gene encodes myoneurin protein, which has been associated with several cancer pathogenesis and disease development processes. However, there needed to be a more detailed characterization of this polymorphism’s (and other coding and non-coding polymorphisms) structural, functional, and molecular impact. The current study addressed this gap and analyzed different properties of rs10936599 and non-coding SNPs of MYNN via a thorough computational method. The variant, rs10936599, was predicted functionally deleterious by nine functionality prediction approaches, like SIFT, PolyPhen-2, and REVEL, etc. Following that, structural modifications were estimated through the HOPE server and Mutation3D. Moreover, the mutation was found in a conserved and active residue, according to ConSurf and CPORT. Further, the secondary structures were predicted, followed by tertiary structures, and there was a significant deviation between the native and variant models. Similarly, molecular simulation also showed considerable differences in the dynamic pattern of the wildtype and mutant structures. Molecular docking revealed that the variant binds with better docking scores with ligand NOTCH2. In addition to that, non-coding SNPs located at the MYNN locus were retrieved from the ENSEMBL database. These were found to disrupt the transcription factor binding regulatory regions; nonetheless, only two affect miRNA target sites. Again, eight non-coding variants were detected in the testes with normalized expression, whereas HaploReg v4.1 unveiled annotations for non-coding variants. In summary, in silico comprehensive characterization of coding and non-coding single nucleotide polymorphisms of MYNN gene will assist researchers to work on MYNN gene and establish their association with certain types of cancers.

## Introduction

Single nucleotide polymorphisms (SNPs) are the most prevailing forms of genome variation in the human genome, where multiple alleles can exist in some population(s), and the frequency of the least common allele must be at least 1%. They occur approximately every 300–400 base pairs away [1]. It has been reported that SNPs are associated with disease markers, disease susceptibility, and genomic evolution [2]. A high-throughput molecular biology technique called a genome-wide association study (GWAS) sheds light on the relationship between the frequency of single-nucleotide polymorphisms (SNPs) and other forms of genetic variants and specific phenotypes. In recent years, GWAS has led to the discovery of numerous genetic loci or regions associated with common diseases, including cancers [3, 4]. GWAS Catalog [5] has revealed that a non-synonymous polymorphism (rs10936599) at chromosome 3q, covering the MYNN gene, is correlated with colorectal cancer [6], telomere length [7], multiple myeloma [8], bladder cancer [9], and so on.

MYNN gene, located on the 3q26.1 chromosome, encodes a 610 amino acids long protein called myoneurin (isoform A) [10]. This protein mainly functions as a transcriptional repressor and belongs to the POK (Poxviruses and Zinc-finger (POZ) and Krüppel) family [11]. It is categorized by the existence of an amino-terminal POZ/ Broad Complex, Tramtrack, and Bric a’ brac (BTB) domain in addition to eight Kruppel-type zinc fingers at the carboxy-terminal moiety [10, 11]. The BTB/POZ domain mediates protein-protein interactions with transcriptional co-factors (corepressors, histone deacetylases) through homo-dimerization and hetero-dimerization. The recruitment of transcriptional corepressors and histone deacetylases induces heterochromatin formation, followed by inhibition of transcription activation. However, Krüppel-type zinc finger motifs are responsible for the DNA binding properties. This gene is associated with gene expression, cancer development, and tumorigenesis [11]. Additionally, it regulates BMP signaling [12], synaptic gene expression [13], skeletal muscle growth [10], etc.

Reportedly, rs10936599 is associated with shorter telomere lengths and biological ageing [14]. Moreover, there may be a significant correlation between the polymorphisms for Telomerase RNA Component (TERC) (rs2293607) and MYNN (rs10936599), which is responsible for elevated risk of colorectal cancer, colorectal adenomas [15], and bladder cancer [16]. Additionally, it has also impacted the elevated hazard of chronic obstructive diseases [17], chronic lymphocytic leukemia [18], cutaneous melanoma [19], and multiple sclerosis [20], etc. Despite the clinical significance of rs10936599, the molecular functions and structural mechanisms are not fully established yet. This study aimed to detect the effect of this single nucleotide polymorphism on the functional characteristics, structural mechanisms, and dynamic behavior of myoneurin protein. The insights of this study can contribute to the research and development of personalized treatments and medications.

## Materials and methods

### Retrieval of Non-synonymous SNPs (nsSNPs)

MYNN gene was selected for in-silico analysis from the literature study as it has been reported to be associated with several cancer development processes [8, 16, 18]. Then, we investigated the human MYNN gene in the ENSEMBL genome browser [21] (https://asia.ensembl.org/index.html) and selected the ENST00000349841.10 transcript encoding 610 amino acids long myoneurin protein. Missense variants were filtered using the global minor allele frequency (MAF) value (0.05–0.5). Moreover, the protein sequence was retrieved from UniProt [22] (https://www.uniprot.org/).

### Functional consequence analysis of nsSNPs

**Sort Intolerant From Tolerant (SIFT)** (https://sift.bii.a-star.edu.sg/) was employed to detect the deleteriousness of nsSNPs. SIFT can distinguish the deleterious and neutral effects of amino acid substitutions in nsSNPs and missense mutations based on physical characteristics and sequence homology of amino acids [23]. It utilizes multiple sequence alignment to obtain normalized probability scores for all substitutions. A score <0.05 is considered a deleterious substitution.

**Polymorphism Phenotyping v2 (PolyPhen-2)** (http://genetics.bwh.harvard.edu/pph2/) is a publicly accessible web server for predicting the structural and functional consequences of amino acid substitutions [24]. Variants with PolyPhen-2 score of (0.0–0.15) are considered benign, (0.15–1.0) as possibly damaging, and (0.85–1.0) as damaging.

**The Rare Exome Variant Ensemble Learner (REVEL)** (https://sites.google.com/site/revelgenomics/) is an ensemble method for detecting the pathogenic nsSNPs based on tools, namely MutPred, PolyPhen, FATHMM, SIFT, MutationAssessor, PROVEAN, and several ensemble methods. REVEL score ranges from (0–1) with a cut-off of 0.5 [25].

**MetaLR** (https://wglab.org/) distinguishes between neutral and damaging SNPs using logistic regression by providing a score between 0 to 1, where a score>0.5 indicates the damaging effect [26]. **MutationAssessor** (http://mutationassessor.org/r3/) is a web server that estimates the functional effect of missense polymorphisms and mutations based on evolutionary conservation in protein homologs. It produces a score ranging from 0 to 1. nsSNPs with higher scores are more likely to be pathogenic [27].

**MutPred2** (http://mutpred.mutdb.org/), a machine learning-based method, estimates the pathogenicity and molecular alteration of single nucleotide polymorphisms by integrating genetic and molecular data [28]. MutPred2 generates a general score from the mean scores of the neural networks. A score cut-off of 0.50 denotes pathogenicity. **Protein ANalysis THrough Evolutionary Relationships (PANTHER)** (http://www.pantherdb.org/tools/) is a comprehensive, freely available database that employs phylogenetics to analyze protein sequences and determine their evolutionary links to other proteins [29]. It employs PANTHER-PSEP (Position-Specific Evolutionary Preservation) to anticipate how nonsynonymous coding single nucleotide polymorphisms may affect the functionality of proteins [30].

**ClinVar** (https://www.ncbi.nlm.nih.gov/clinvar/) is a public database of genetic variants and their clinical significance that gathers data from a variety of sources, such as clinical testing facilities, research projects, and the scientific literature, and disseminates knowledge regarding the associations between genetic variants and diseases or other health issues [31]. **PON-P2** (http://structure.bmc.lu.se/PON-P2/) is a machine learning-based tool that has been developed for the classification of amino acid substitutions in human proteins, utilizing the evolutionary conservation of sequences, the physical and biochemical properties of amino acids, Gene Ontology (GO) annotations, and functional annotations of variation sites [32].

### Protein-protein interaction

**NetworkAnalyst** (https://www.networkanalyst.ca/) was employed for predicting protein-protein interaction. With the aid of NetworkAnalyst, generic PPI networks, cell-type or tissue-specific PPI networks, gene regulatory networks, gene co-expression networks, networks for toxicogenomics and pharmacogenomics studies, and networks for gene co-expression profiling can be built [33]. Additionally, gene ontology (biological process, molecular function, and cellular component) data were retrieved from NetworkAnalyst, and the gene ontology plot was generated using the ggplot2 package in R programming [34].

### Structural analysis

To analyze the structural impact of missense variants, we used the **HOPE** web tool [35] (https://www3.cmbi.umcn.nl/hope/), an automatic mutant server. It integrates data from various sources, namely genetic annotations from the UniProt database, prediction models from DAS services, protein’s structural coordinates from WHAT IF web services, and homology models from YASARA.

**Mutation3D** (http://www.mutation3d.org/) is a new algorithm and web server that uses a 3D clustering approach to analyze the distribution of amino acid substitutions within tertiary protein structures [36]. **ConSurf** [37] (https://consurf.tau.ac.il/consurf_index.php) is a publicly accessible bioinformatics tool to estimate the evolutionary conservancy of amino acid substitution, using either an empirical Bayesian method [38] or a maximum likelihood method [39]. The conservation scores provide a relative indicator of evolutionary conservation, where the lowest conservation score denotes the most conserved position in the sequence. The analysis was carried out with the default parameters.

**CPORT (Consensus Prediction Of interface Residues in Transient complexes)** (https://alcazar.science.uu.nl/services/CPORT/) is a consensus method that combines six interface prediction web servers to predict interface residues in protein-protein complexes [40]. It generates more stable and reliable predictions than individual predictors alone and competitive results with the *ab initio* methods. CPORT was employed to detect the active residues in protein-protein or protein-ligand complexes.

### Secondary structure prediction

**SOPMA (Self-Optimized Prediction method With Alignment)** (https://npsa-prabi.ibcp.fr/cgi-bin/npsa_automat.pl?page=/NPSA/npsa_sopma.html), a bioinformatics tool, was utilized for predicting the secondary structure of the protein [41]. Based on the homologue model [42], it generates a secondary structure with 73.2% accuracy.

### 3D structure modeling

**I-TASSER** (https://zhanggroup.org/I-TASSER/), a KU-developed bioinformatics tool for predicting protein structure, was used to model tertiary structure [43]. Based on the significance score of various threading templates and clustering density, the program calculates the C-score to measure the accuracy of the predictions. The produced structures were refined using **GalaxyWEB** (https://galaxy.seoklab.org/cgi-bin/submit.cgi?type=REFINE) [44]. It is a server for refining protein structures based on the ab initio method.

### Structural models assessment

The improved structures were validated by several structure validation programs, such as **PROCHECK (SAVES v6.0)** [45] (https://saves.mbi.ucla.edu/), **ProSA-web** [46] (https://prosa.services.came.sbg.ac.at/prosa.php), and **Structure Assessment—SWISS-MODEL** [47] (https://swissmodel.expasy.org/assess). A protein structure can be evaluated for its stereochemical quality using the PROCHECK suite. Besides, Z-score is displayed by the ProSA tool (Protein Structural Analysis) for model evaluation.

Further, RMSD and TM-score between the wildtype and variant structure were estimated using **TM-align** (https://zhanggroup.org/TM-align/), a bioinformatics tool for protein sequence alignment [48] and **pyMOL** [49] (https://pymol.org/2/).

### Molecular docking

The mutant and the wildtype structure were subjected to molecular docking with a target protein. As a negative control, two independent ligands were also docked against these protein structures. The docking was performed using the **HDOCK** server [50] (http://hdock.phys.hust.edu.cn/). This server is designed to estimate the protein-protein or protein-nucleic acid binding complexes based on a hybrid approach of ab initio and template-based modeling. The predicted complexes were visualized using **PyMOL** and **Biovia Discovery Studio** [51] (https://discover.3ds.com/discovery-studio-visualizer-download).

### Molecular dynamics

**GROMACS (version 2020.6)** simulation software (https://www.gromacs.org/) was employed to conduct 100 nanoseconds Molecular Dynamics simulations for both the wildtype and variant models [52]. The simulation chose GROMOS96 43a1 force-field. The spc216 water model was deployed to build a water box with edges of 0.5 nm from the protein surface. We employed the proper ions to balance the systems. Following energy minimization, isothermal-isochoric (NVT) equilibration, and isobaric (NPT) equilibration of the system, a 100 nanoseconds molecular dynamics simulation with periodic boundary conditions was carried out. The 100 picoseconds snapshot interval was specified in order to analyze the trajectory data. The GROMACS software’s integrated rms, rmsf, gyrate, and sasa modules were used to perform the root mean square deviation (RMSD), root mean square fluctuation (RMSF), radius of gyration (Rg), and solvent accessible surface area (SASA) studies once the simulation was performed. Using the ggplot2 program in RStudio, the plots for each of these experiments were generated.

### Functional analysis of non-coding SNPs

Non-coding SNPs (introns, 5’ UTR, 3’UTR) were retrieved from the ENSEMBL database by filtering a MAF value of 0.05–0.5. These non-coding SNPs were analysed in **RegulomeDB** (https://regulomedb.org/regulome-search), a database that provides comprehensive annotation of genetic variants in the non-coding regions of the human genome [53]. Furthermore, the annotated SNPs proceeded for analysis in **GTEx Portal** [54] (https://gtexportal.org/home/). The Genotype-Tissue Expression (GTEx) project is an extensive free-access repository to study tissue-specific gene expression and regulation.

Moreover, the functional importance of the non-coding SNPs was detected by employing **HaploReg v4.1** [55] and **PolymiRTS** [56]. HaploReg (https://pubs.broadinstitute.org/mammals/haploreg/haploreg.php) is a publicly accessible bioinformatics tool to investigate non-coding genomic annotations at variations on haplotype blocks, like potential regulatory SNPs at genetic disorder loci. The polymorphism in microRNA target site (PolymiRTS) (https://compbio.uthsc.edu/miRSNP/) is a comprehensive database that provides information about genetic polymorphisms (SNPs) in microRNAs (miRNAs) and their target sites.

**Fig 1:**
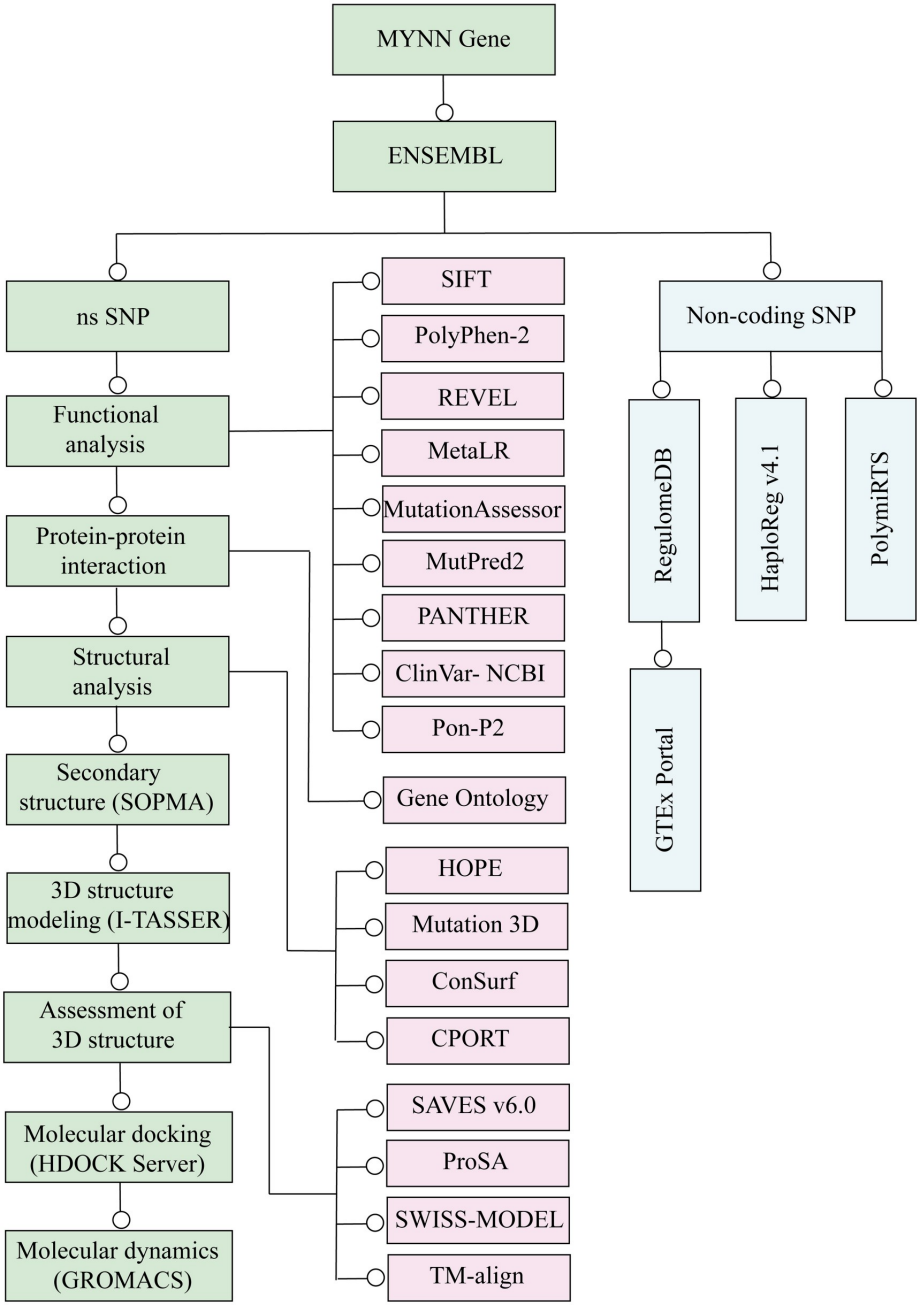
Flowchart of the pipeline of the analysis.

A schematic representation of the workflow of this study is provided in Fig 1.

## Results

### nsSNP data retrieval

From the ENSEMBL database, only one nsSNP (rs10936599) was obtained from the ENST00000349841.10 transcript with a MAF value of 0.27. Interestingly, this particular SNP has also been found for the MYNN gene in the GWAS Catalog [5], a curated genome-wide association study database. In this analysis, we focused on the G allele of this variant, where histidine is replaced with glutamine at position 6.

### Results of functional consequence prediction

**Fig 2:**
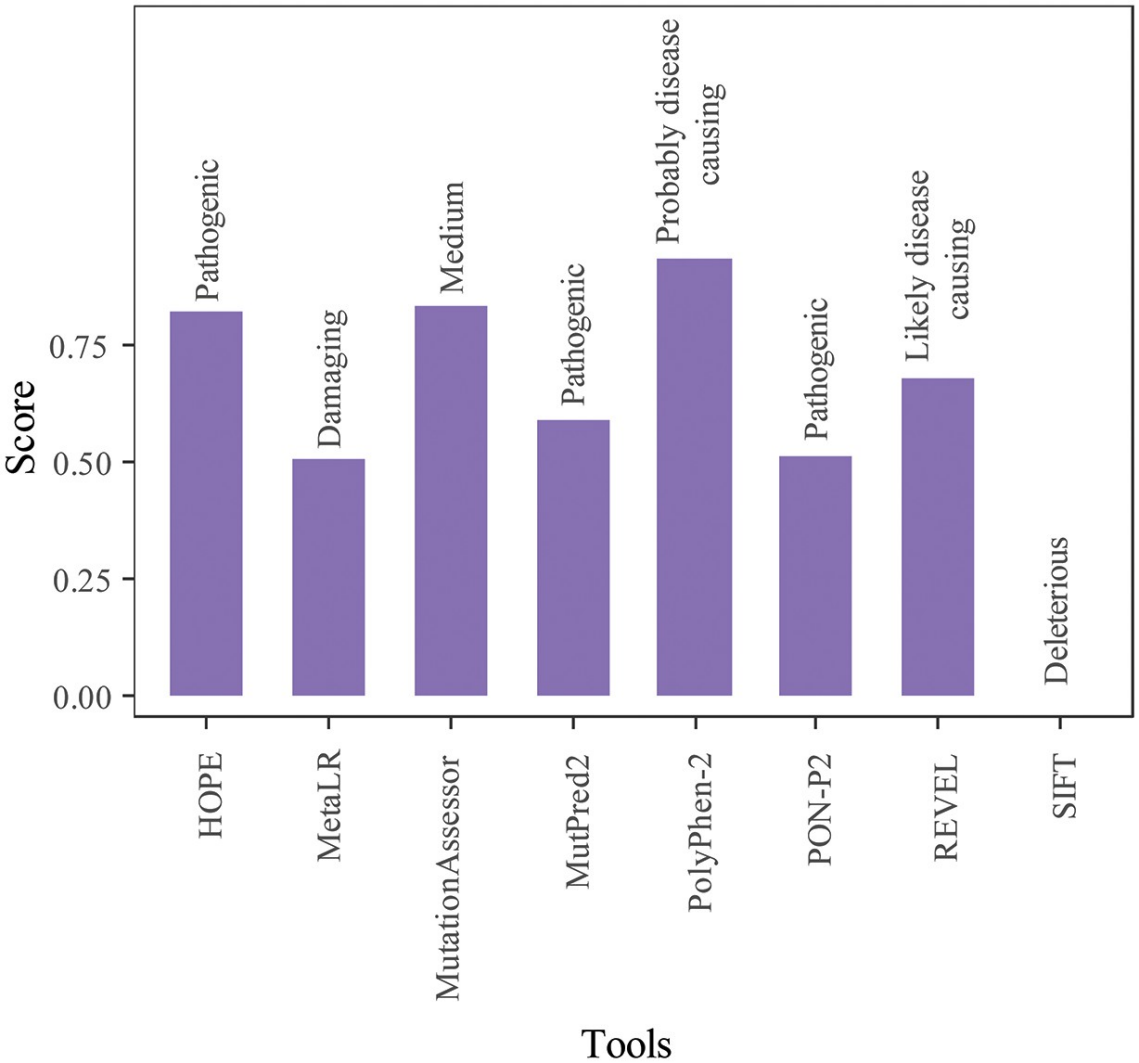
Deleterious effect of rs10936599 in several web tools.

The functional impact of rs10936599 was assessed in nine bioinformatics-based web tools. All these tools predicted that this specific amino acid substitution at position 6 affects the function of myoneurin protein (Table 1). The prediction scores of these tools are represented in Fig 2.

**Table 1:** Functional predictions of rs10936599 from nine bioinformatics tools with threshold levels.

Tools	Threshold Level	Score	Prediction	URL
SIFT	<0.05	0	Deleterious	https://sift.bii.a-star.edu.sg/
PolyPhen-2	>0.95	0.94	Probably damaging	http://genetics.bwh.harvard.edu/pph2/index.shtml
REVEL	>0.5	0.68	Likely disease causing	https://sites.google.com/site/revelgenomics/
MetaLR	>0.5	0.51	Damaging	https://wglab.org/members/15-member-detail/36-coco-dong
MutationAssessor	Functional impact score> 3.5 (High) 1.9<Functional impact factor<3.5 (Medium)	0.838	Medium	http://mutationassessor.org/r3/
MutPred2	>0.5	0.593	Pathogenic	http://mutpred.mutdb.org/index.html
PANTHER	Time > 450my (Probably damaging) 450my > Time > 200my (Possibly damaging)	325	Possibly damaging	http://www.pantherdb.org/
ClinVar- NCBI			Associated	https://www.ncbi.nlm.nih.gov/clinvar/
PON-P2	>0.5	0.512	Pathogenic	http://structure.bmc.lu.se/PON-P2/
HOPE	>0.5	0.827	Pathogenic	https://www3.cmbi.umcn.nl/hope/

### Analysis of MYNN (myoneurin) interaction

**Fig 3:**
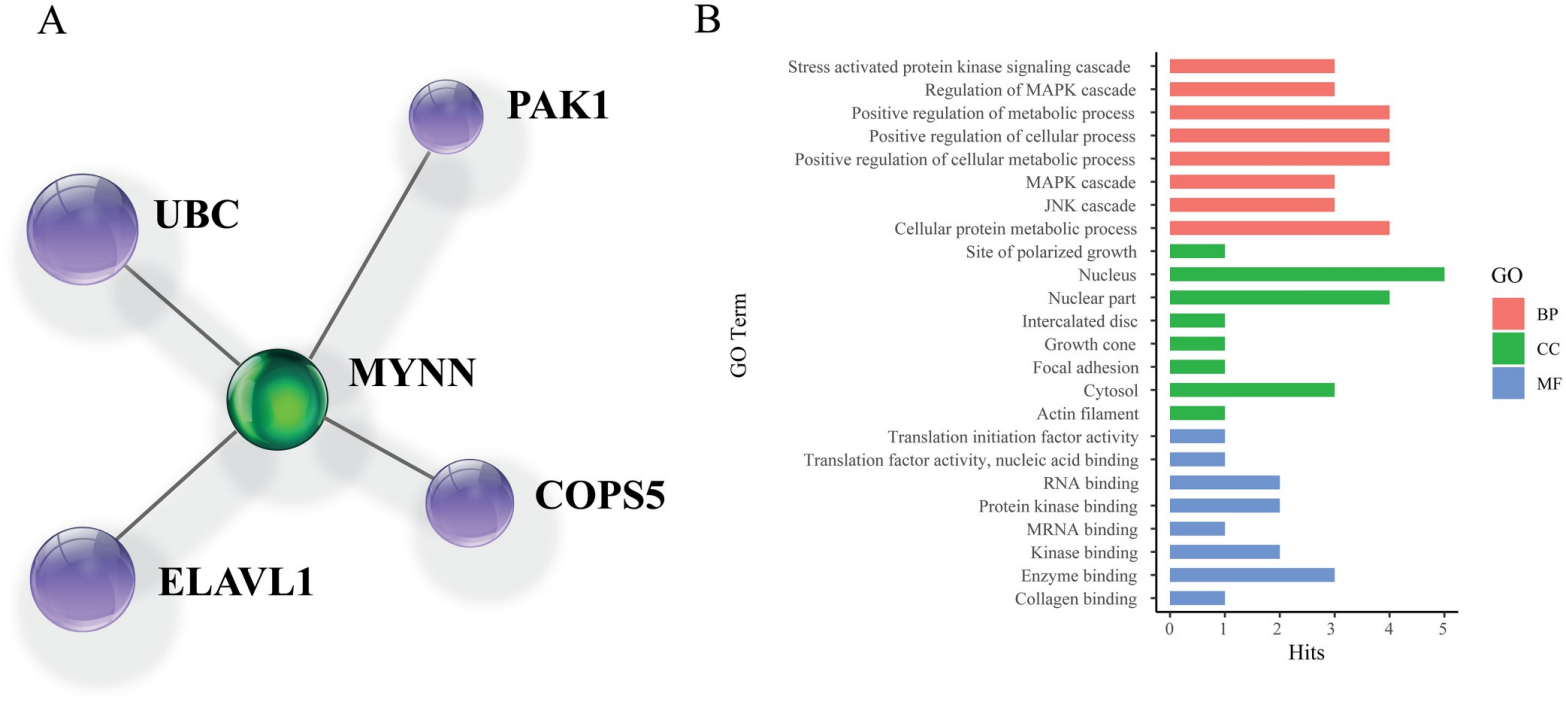
A) Interaction of MYNN with other cellular proteins. B) Significant GO terms associated with MYNN.

NetworkAnalyst demonstrated that four proteins (UBC, PAK1, COPS5, and ELAVL1) interact with MYNN (Fig 3). These proteins are associated with numerous pathways, including gene expression, regulatory processes, cancer development, and cancer metastasis. It also revealed that this gene is significantly associated with 63 biological pathways, including the JNK cascade, MAPK cascade, cellular metabolic processes, hypoxia, etc. (S1 Table). Regarding molecular function, MYNN is involved in enzyme binding, kinase binding, nucleotide binding, etc. The most significant GO terms in cellular components are the nucleus, cytosol, sarcomere, etc. The top significantly enriched terms of biological process, molecular function, and cellular components of gene ontology analysis are visualized in Fig 3.

### Effect of rs10936599 on the structure of the protein

#### Analysis of structural modifications

**Fig 4:**
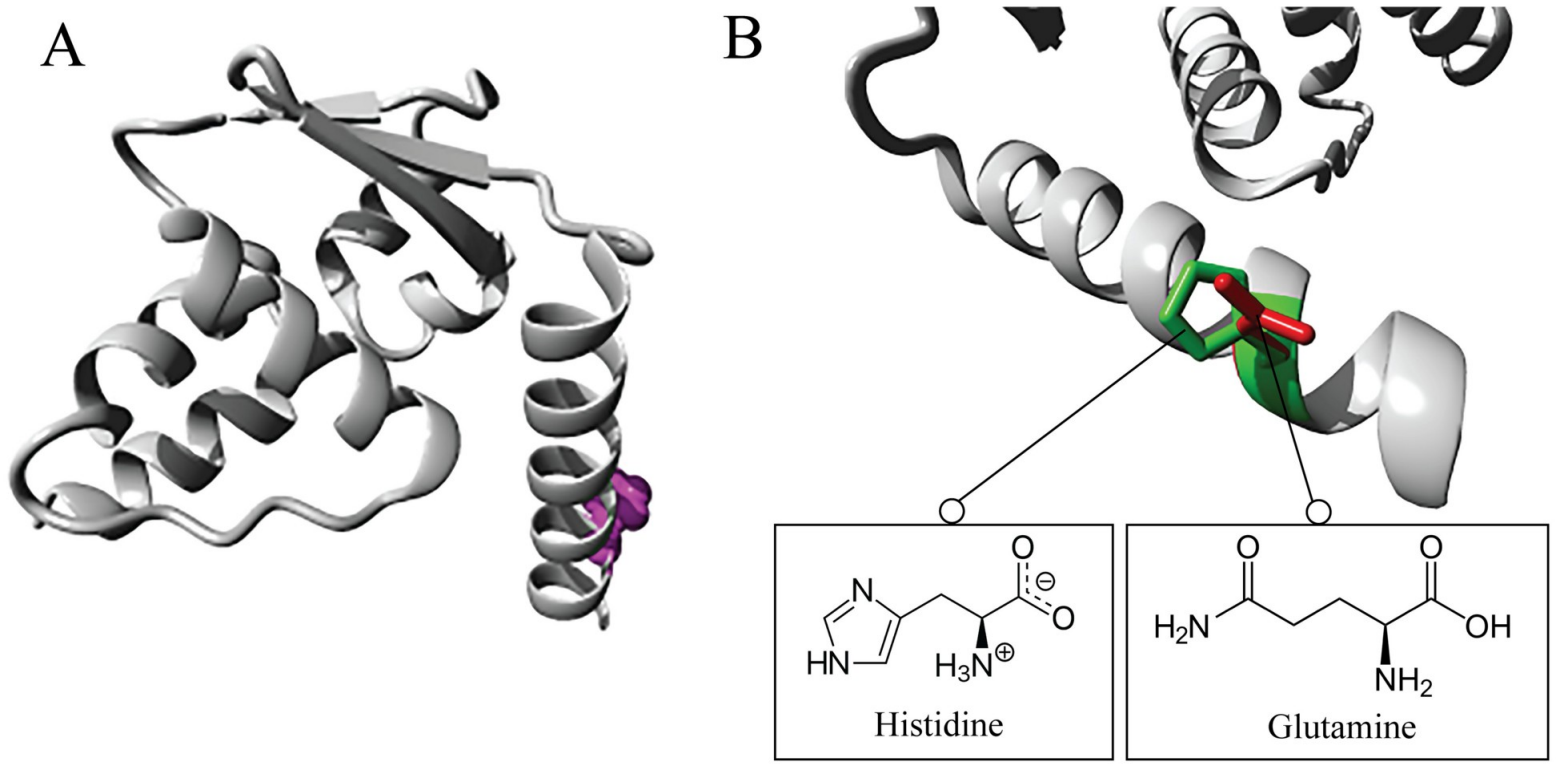
**A)** Demonstration of the protein used in ribbon display. The side chain of the mutant residue is represented as little balls and is highlighted magenta, along with the protein, which is highlighted grey. **B)** Close-up of the substitution, where the protein is shaded grey along with the side of wildtype and mutant amino acid in green and red, respectively.

Amino acid substitution from histidine to glutamine at position 6 was checked in the HOPE server. This server predicted that the variant residue is smaller than the wildtype, which can affect potential external interactions. Also, the wildtype amino acid seems highly conserved at this position, and this particular mutant residue is not present in homologous proteins. It suggests that the variant hardly results without affecting the protein. Furthermore, the MetaRNN score of the substitution is 0.827, indicating that rs10936599 is more likely to be pathogenic. The altered residue is found outside a domain without known function and nearby Skp1/Btb/Poz Domain Superfamily. This residue rarely interacts with any known domain but potentially affects interaction with others. The 3D structure gathered by the HOPE server is represented in Fig 4.

#### Domain identification in tertiary structure

Mutation 3D revealed that myoneurin protein consists of two known domains: BTB domain and zf-H2C2_2 domain. BTB domain, involved in transcription regulation, ion channel, cytoskeleton dynamics, etc. [57], spans from position 14 to 118. Nevertheless, the other domain, a zinc finger associated with cancer development [58], stretches from amino acid 372 to 398. Additionally, our mutation of interest was found proximal to the BTB domain.

#### Analysis of conservancy and active residues of the protein

**Fig 5:**
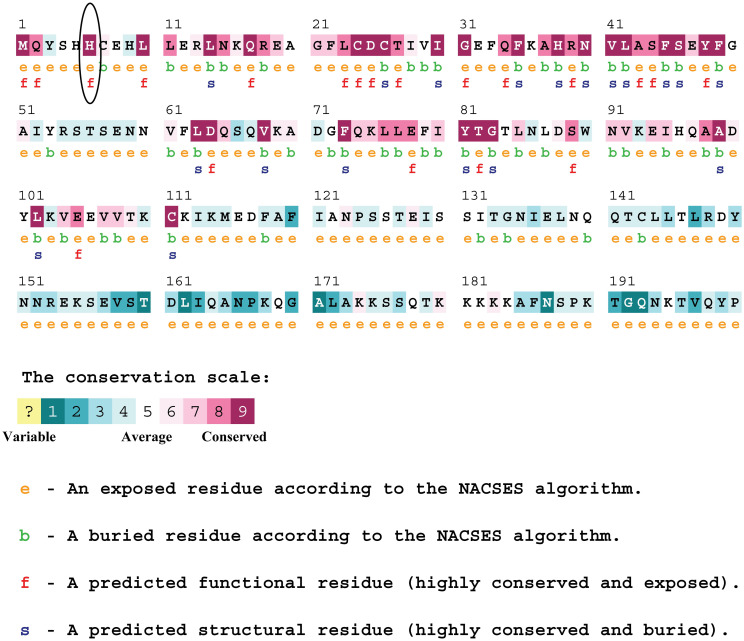
Visualization of conservational analysis in ConSurf.

According to ConSurf, position 6 of the MYNN protein sequence is a highly conserved, exposed, and functional residue (Fig 5). It indicates that polymorphism at this position is deleterious for the function and structure of the protein. CPORT also disclosed that position is among the active residues of the protein.

### Impact of rs10936599 on protein secondary structure

SOPMA unveiled the comparative secondary structures of wildtype and nsSNP (S1 Fig). The wildtype structure consists of 30.16% (184 residues) alpha helix, followed by 16.39% (100 residues) extended strand and 6.89% (42 residues) beta-turn. However, the variant structure contains 30.66% (187 residues), 16.56% (101 residues), and 6.23% (38 residues) of alpha helix, extended strand, and beta-turn, respectively. Both of the structures contain 46.56% (284 residues) random coil. Also, the substituted amino acid is located at the alpha helix region. Apparently, there is a difference in both structures, which might cause some functional differences.

### Tertiary structure analysis through model simulation

**Fig 6:**
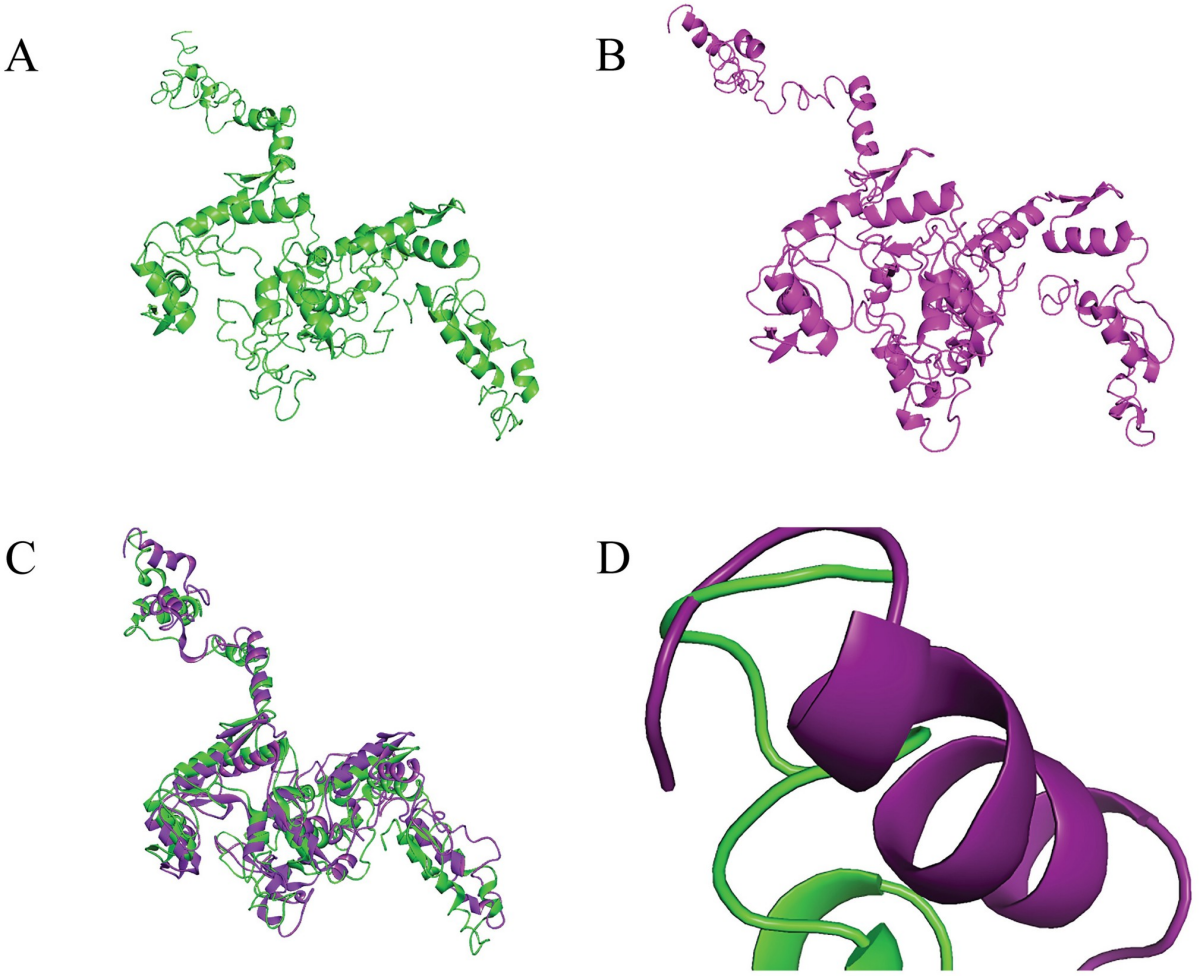
**A)** Model structure of wildtype protein. **B)** Model structure of nsSNP protein. **C)** Superimposed display of wildtype and variant structure, where wildtype is colored in green and variant in purple. **D)** Superimposition of the mutated amino acid position in both models. The wildtype structure is shaded in green and nsSNP in purple.

I-TASSER generated tertiary structures for wildtype and nsSNP, using fold recognition or protein threading method with C scores of -3.78 and -3.91, respectively (Fig 6). Usually, the C-score lies between [–5,2], where a higher C score implicates higher confidence [43].

Post refinement in GalaxyWEB, these models were evaluated in PROCHECK, ProSA, Structure Assessment—SWISS-MODEL, TM-align, and PyMOL. Several quantitative scores from these tools are listed in Table 2. Scores of Ramachandran favored regions are 81.1% and 80.7% for the wildtype and variant models. The Ramachandran plots and Z-score plots for both the native and variant models are provided in S2 Fig. Notedly, there has not been found any template or structure for myoneurin protein in RCSB PDB [59] or any other database. Hence, I-TASSER couldn’t fulfill all requirements for protein threading. However, the RMSD value between the two models is 5.968, which implies a significant deviation between both structures. Moreover, the TM-score of 0.84197 indicates structures are roughly in the same topology.

**Table 2:** Quantitative scores for evaluating modeled structures.

ID	Ramachandran favored region	PROSA Z-score	MolProbity Score	RMSD	TM-score
**Wild Type**	81.1	-4.12	1.93	5.968	0.84197
**rs10936599**	80.7	-4.3	1.85		

### Molecular docking analysis

**Fig 7:**
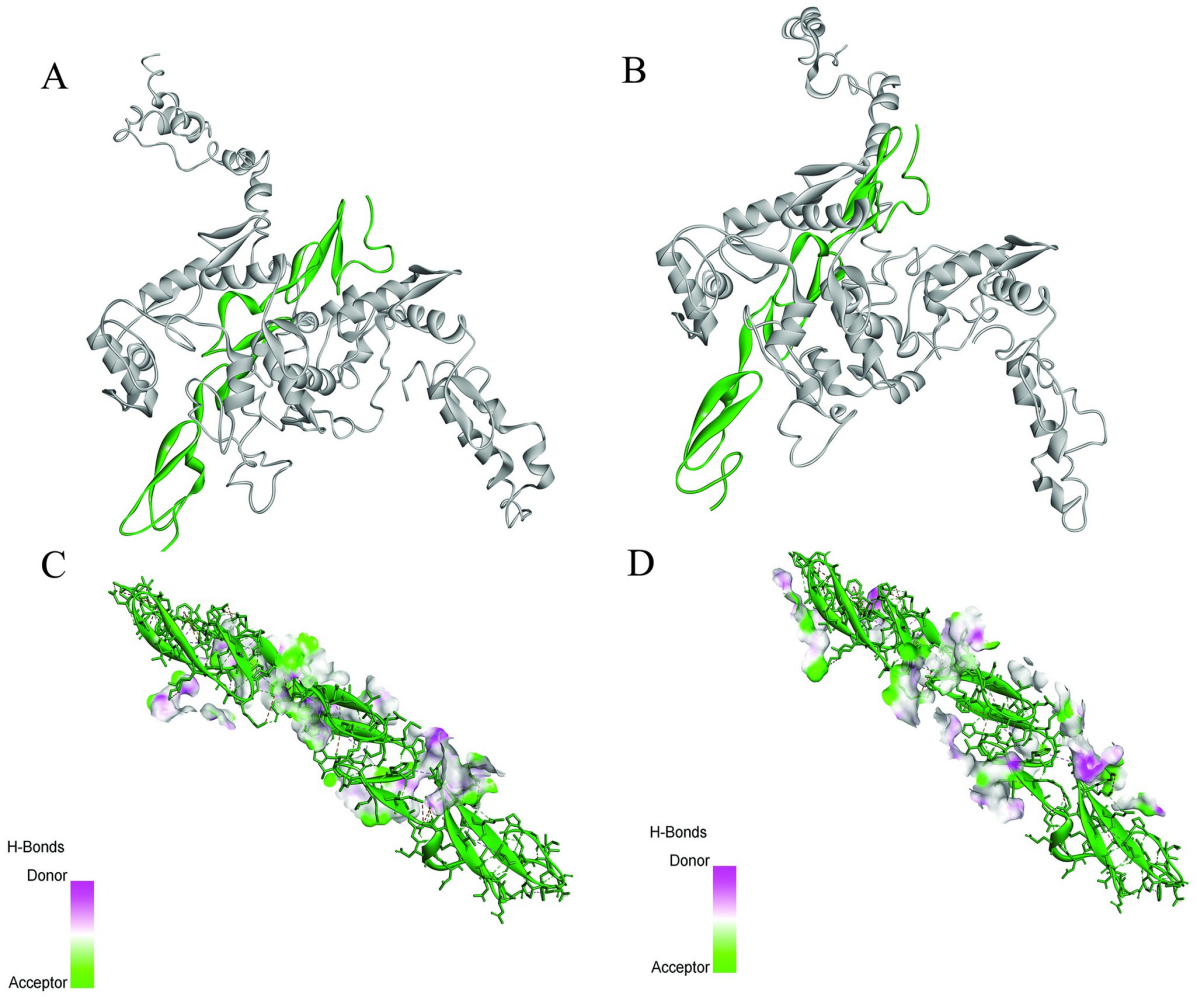
Visualization of the molecular docking complexes of **A)** wildtype with NOTCH2 **B)** nsSNP with NOTCH. Here, variant and wildtype structures are shaded in grey, whereas NOTCH is highlighted in green. Ligand interactions in **C)** wildtype and NOTCH2 complex **D)** variant and NOTCH2 with hydrogen bond donor/acceptor surface.

Potential ligands for MYNN were retrieved from several databases [22, 60] and literature studies [61–63]. It was found that NOTCH2 potentially interacts with MYNN [63]. Hence, MYNN protein (myoneurin) was subjected to blind docking to estimate the change in protein-protein interaction. The PDB structure of NOTCH2 was retrieved from RCSB PDB under 5MWB PDB ID. Following docking, the top 10 models for each complex were generated in the HDOCK server. From those models, two compatible models were selected for comparison (Fig 7).

The docking results revealed that the docking scores for wildtype and mutants are -254.7 and -269.55, with confidence scores of 0.8901 and 0.9161, respectively (Table 3). It implies that the mutant binds with NOTCH2 with a higher affinity than the wildtype protein. Additionally, two independent ligands (Acetaminophen and Adderall) were docked with wildtype and variant structures to detect whether these models form non-specific interactions with random ligands. These ligands showed poor docking scores with low confidence scores, indicating these ligands are unlikely to bind with both protein structures.

**Table 3:** Docking scores for all docking complexes.

Ligand	Wild-type		rs10936599	
	Docking Score	Confidence Score	Docking Score	Confidence Score
NOTCH2	-254.7	0.8901	-269.55	0.9161
**Independent Ligand**				
Acetaminophen	-85.41	0.2158	-87.92	0.2242
Adderall	-69.65	0.1670	-82.61	0.2062

### Analysis of dynamic characteristics

Root Mean Square Deviation (RMSD) is calculated to assess the systems’ stability. A higher RMSD value indicates the unstable nature of the protein. The variant seemed to stabilize the protein structure here since the wildtype had a greater RMSD than the variant.

The regional flexibility of the protein is evaluated using the Room Mean Square Fluctuation (RMSF) method. The flexibility of a specific amino acid site increases with RMSF. Compared to the variant MYNN, the residues in the wildtype MYNN protein were generally more flexible.

The degree of compactness is measured by using the radius of gyration. Protein folding is stable when the radius of gyration is relatively constant. The radius of gyration fluctuation implies protein unfolding. With the mutant protein, the radius of gyration drastically decreased, suggesting that it folded quickly. The wildtype MYNN, on the other hand, had a much larger gyrating radius.

**Fig 8:**
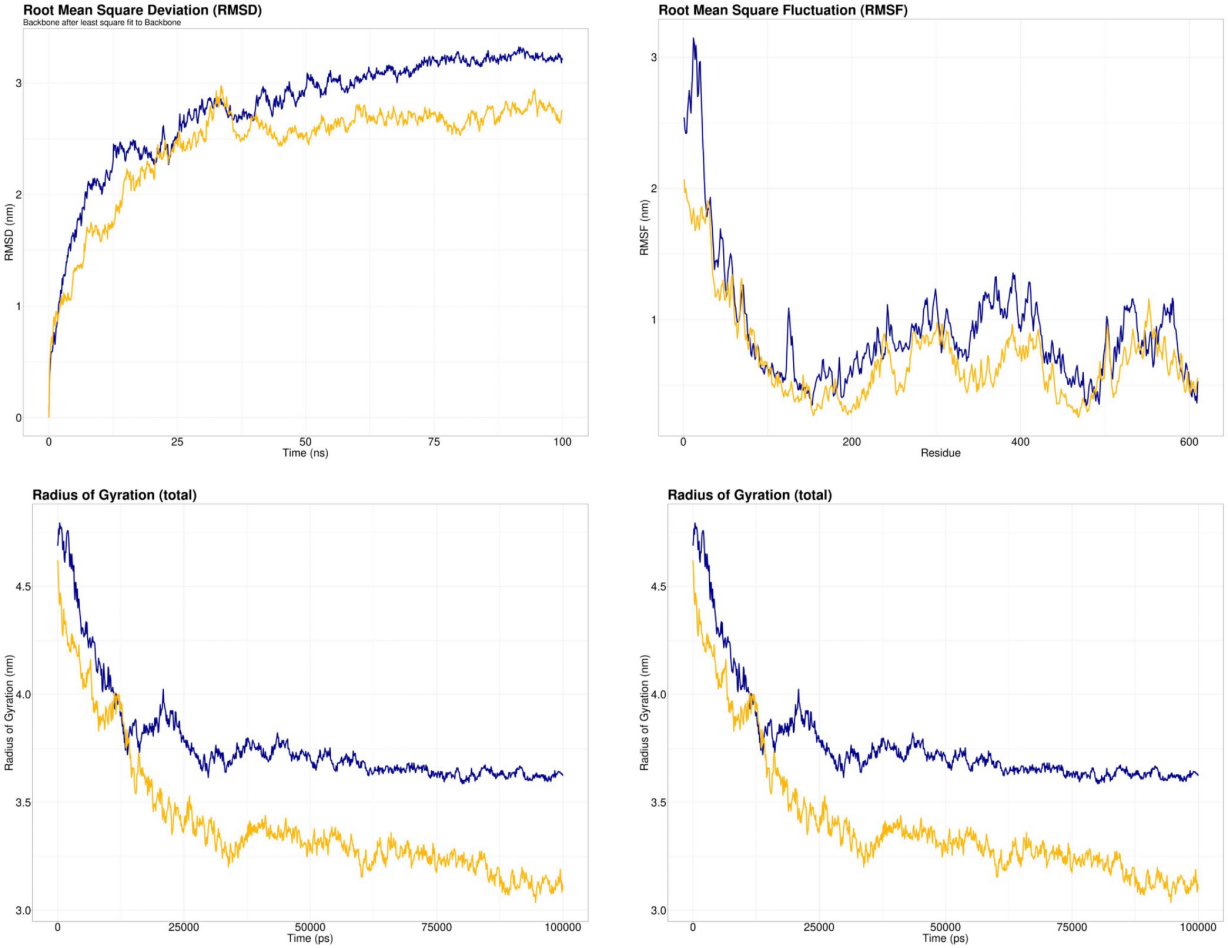
RMSD, RMSF, Radius of gyration, and SASA analysis of wildtype MYNN (blue) and variant MYNN (yellow) protein following molecular dynamic simulations.

In MD simulations, Solvent Accessible Surface Area (SASA) anticipates the stability of proteins’ hydrophobic cores. The probability of protein instability due to solvent accessibility increases with increasing SASA score. SASA levels were higher in the wildtype MYNN than in the variant structure. The results of MD simulations are presented in Fig 8.

### Analysis of functional consequences of non-coding SNPs

A total of 18 non-coding SNPs were retrieved from ENSEMBL. Among them, 14 were intron variants, and four were 3 prime UTR variants (S2 Table).

**Fig 9:**
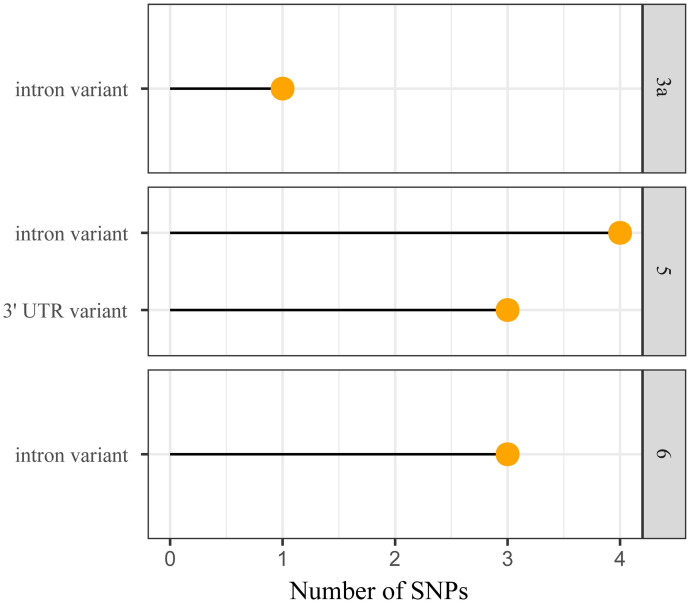
Demonstration of the number of non-coding SNPs located in various regulome ranks. Here, 3a, 5, and 6 denote TF binding + any motif + DNase peak, TF binding or DNase peak, and motif hit, respectively.

RegulomeDB generated regulome ranks and regulome scores for these polymorphisms to predict the functionality of these SNPs (Fig 9). Most of these SNPs were located at transcription factor binding or DNase peak (Rank 5), followed by motif hit (Rank 6) and transcription factor binding + any motif + DNase peak (Rank 3a).

These SNPs proceeded for further analysis in GTEx Portal. Among these, eight SNPs were detected at the testis with normalized effect sizes ranging from 0.28–0.35 (Table 4). Single tissue Expression quantitative trait loci (eQTL) violin plots are illustrated in S3 Fig. Notedly, other genes also showed tissue-specific eQTLs other than MYNN.

**Table 4:** Single tissue eQTL prediction for non-coding SNPs.

Gencode Id	Gene Symbol	Variant Id	SNP Id	P-Value	NES	Single Tissue eQTL
ENSG00000085274.15	MYNN	chr3_169773941_T_A_b38	rs2251795	3.20E-09	0.3	Testis
ENSG00000085274.15	MYNN	chr3_169775495_C_G_b38	rs3950296	2.10E-11	0.35	Testis
ENSG00000085274.15	MYNN	chr3_169776839_A_G_b38	rs9866116	1.90E-09	0.3	Testis
ENSG00000085274.15	MYNN	chr3_169779797_A_G_b38	rs1317082	2.10E-11	0.35	Testis
ENSG00000085274.15	MYNN	chr3_169782699_G_A_b38	rs3772190	2.10E-11	0.35	Testis
ENSG00000085274.15	MYNN	chr3_169785644_C_T_b38	rs2141595	5.30E-09	0.29	Testis
ENSG00000085274.15	MYNN	chr3_169788353_C_T_b38	rs1920122	4.60E-08	0.28	Testis
ENSG00000085274.15	MYNN	chr3_169788385_C_T_b38	rs1920123	4.60E-08	0.28	Testis

These non-coding single nucleotide polymorphisms were assessed in PolymiRTS to detect if these amino acid substitutions affect any miRNA target site. Only two SNPs (rs1920123 and rs75277808) were unveiled to affect miRNA regions. rs1920123 seems to disrupt a conserved target site, whereas rs75277808 happens to create a novel target site.

HaploReg v4.1 was employed to analyze non-coding genomic annotations at variants. Annotations for a total of 11 variants were discovered for the MYNN gene. Among them, eight were intronic variants, and the remaining three were 3’-UTR variants. Annotations for all of these SNPs are reported in Table 5.

**Table 5:** Genomic annotations for non-coding SNPs.

**chr**	3	3	3	3	3	3	3	3	3	3	3
**pos (hg38)**	1.7E+08	1.7E+08	1.7E+08	1.7E+08	1.7E+08	1.7E+08	1.7E+08	1.7E+08	1.7E+08	1.7E+08	1.7E+08
**variant**	rs1317082	rs1881966	rs1920120	rs1920122	rs1920123	rs2141595	rs2251795	rs3772190	rs3950296	rs75277808	rs9866116
**Ref**	A	G	T	C	C	C	T	G	C	G	A
**Alt**	G	A	C	T	T	T	A	A	G	A	G
**AFR freq**	0.07		0.4	0.4	0.4	0.55	0.07	0.07	0.07	0.1	0.57
**AMRf req**	0.29		0.36	0.36	0.36	0.36	0.33	0.29	0.29	0.04	0.36
**ASN freq**	0.58		0.69	0.69	0.7	0.7	0.59	0.58	0.58	0.11	0.69
**EUR freq**	0.25		0.27	0.27	0.27	0.27	0.29	0.25	0.25	0.01	0.27
**Promoter histone marks**							23 tissues				
**Enhancer histone marks**	BLD, BRN	BRST, BLD, GI							21 tissues		BLD, GI
**DNAse**							6 tissues				
**Proteins bound**							HAE2F1				
**Motifs changed**	5 altered motifs	4 altered motifs	4 altered motifs	Evi-1	4 altered motifs		CEBPA	GATA,RXRA,TATA	Mrg,Pbx3	E2A,ZBTB7A,Zfp161	5 altered motifs
**dbSNP func annot**	intronic	intronic	intronic	3’-UTR	3’-UTR	intronic	intronic	intronic	intronic	3’-UTR	intronic

## Discussion

MYNN gene encodes myoneurin protein, which is highly expressed in neuromuscular junctions and involved in regulating muscle attachment and neuromuscular networks [64]. Single nucleotide polymorphism of MYNN, rs109365 has an impact on the telomere length [14, 64], gene expression [11], developmental processes [12], and several cancer development processes [6, 15, 16, 18, 19, 65]. C allele acts as the ancestral allele in rs10936599, whereas minor alleles are the T allele with a global MAF value of 0.27 or the G allele [66]. Previously, it has been reported that the CC genotype entails a higher risk of bladder cancer [9, 16], colorectal cancer [6, 15], and multiple myeloma [8]with higher odds ratios. Nevertheless, the T allele demonstrates a relatively protective polymorphism with decreased odds ratios for bladder cancer [16], colorectal cancer [67], and telomere length [7]. In this study, the objectives were to discover the functional and structural alterations in myoneurin protein owing to rs109365599 (G allele) and how it impacts the susceptibility to associated diseases.

Bioinformatics tools and approaches are preferred for converting large-scale and complicated biological datasets into relevant and valuable information [68] because of the more straightforward and time-saving techniques [69]. To assess the functional impact of nsSNP, a comprehensive analysis was conducted by employing several in silico tools and methods. Each prediction tool uses an exclusive algorithm with a specified degree of precision for locating harmful SNPs, strengthening the prediction analysis. These tools address sequence homology, physiological features, and genetic, molecular, and statistical data and ensure the highest accuracy. A total of nine bioinformatics tools were used for predicting functional alterations, and all of the tools revealed that this amino acid substitution significantly disrupts the normal function of the protein.

For a better comprehension of the significance of the MYNN gene, protein-protein interaction was assessed in NetworkAnalyst. It revealed that myoneurin interacts with ubiquitin C, COP9 Signalosome Subunit 5, P21 (RAC1) Activated Kinase 1, and ELAV-like protein 1. Additionally, gene ontology analysis was performed to categorize the biological processes, cellular components, and molecular functions related to this gene. It was observed that myoneurin, majorly located in the nucleus, is significantly involved in numerous signaling and regulatory pathways, namely the JNK cascade, MAPK cascade, cell cycle, transcription, etc. It’s also linked to biological functions like enzyme binding, transcription regulation, translation initiation, etc. Hence, presence of single nucleotide polymorphisms might disrupt these cellular functions and processes.

Furthermore, to determine the general physiological and functional alterations due to the point mutation, nsSNP was subjected to analysis in the HOPE server. It unveiled that the desired SNP decreases the size of the protein, interrupting external interactions. The amino acid alteration modifies the structure of the protein and suggests this SNP as deleterious. Mutation 3D was employed to investigate the amino acid change in the spatial pattern of protein structure and domain identification. This tool reported mainly 2 domains: BTB domain (11–118) and zf-H2C2_2 domain (372–398). It also unveiled that our concerned mutation is located near the BTB domain.

The evolutionary rate of an amino acid position is significantly affected by its structural and functional relevance. Functionally and structurally critical amino acids are highly conserved because even minor alterations at these residues can cause potential modifications in the protein’s function [37]. ConSurf disclosed that position 6 in wildtype MYNN is highly conserved, exposed, and functional residue. CPORT identified binding site amino acids that interact with the substrate or other proteins. According to CPORT, our mutation of interest was found among the active residues.

Due to the absence of myoneurin tertiary structure in RCSB PDB, 3D structures were predicted using the I-TASSER server, which resulted in C scores of -3.78 and -3.91 for wild type and variant, respectively. It is evident that the C scores were relatively lower for these predicted structures. Considering that the MYNN protein sequence lacks a tertiary structure in RCSB PDB and that the I-TASSER prediction is based on protein threading, these scores seemed reasonable. Moreover, this approach was also used in earlier research to predict the three-dimensional structure of proteins [70, 71]. GalaxyWEB was also employed for the structure refinement process.

The generated structure models were evaluated based on the Ramachandran plot, ERRAT score, MolProbity score, and Z score, produced by PROCHECK, Structure Assessment—SWISS-MODEL, and ProSA. The atomic particles are regarded as solid spheres with van der Waals radii in Ramachandran plots. Any angle that causes sphere collisions is sterically unfavorable; hence, such conformations are disallowed. White areas indicate polypeptide conformations where atoms are closer than their van der Waals radii. These areas are sterically hindered for all amino acids except glycine, which has no side chain. The acceptable alpha-helical and beta-sheet configurations are red since they have no steric conflicts. Yellow sections indicate allowed regions if shorter van der Waals radii are involved in the computation, allowing atoms to gather closely. This reveals a left-handed alpha-helix area [72, 73]. The Ramachandran plot illustrates the protein backbone’s torsional angles (ϕ and ψ), where 90% of residues should be in the most favorable locations [74, 75]. 81.1% and 80.7% residues of native and variant structures were located in the Ramachandran favored region, respectively. These scores are justified in the sense that there is no tertiary structure found for the MYNN protein sequence, and I-TASSER prediction is based on protein threading.

Molprobity is a highly recognized technique for validating protein and nucleic acid tertiary structures. It evaluates structure quality using all-atom contact analysis. Structure quality increases as the score approaches 0 [76]. However, the ProSA Z-score estimates the structure’s overall energy deviation from an arbitrary configuration energy distribution. Z-score of -6.07 indicates model quality [46]. MolProbity scores of 1.93, 1.85, and ProSA Z-scores of -4.12, and -4.3 for native and variant structure models, respectively, suggest these models be acceptable.

The structural deviation between wildtype and missense variant structures was estimated based on TM-score and RMSD values predicted by TM-align and PyMOL consecutively. The root mean square deviation (RMSD) between homologous molecules of two protein chains is a widely utilized estimate of similarities between protein structures. The lower RMSD implicates similar structures [77]. The RMSD value of 5.968 indicates a significant deviation between both models. Again, TM-scores, another measure of protein similarity, range from 0 to 1, with 1 indicating a perfect match between two structures, below 0.2 implicating a random match, and above 0.5 presuming roughly the same fold [78]. TM-score of 0.84197 suggests that not only there is a significant deviation between structures, but also they are not randomly matched. Again, the secondary structure prediction by SOPMA also disclosed the difference between mutant and native models.

Molecular docking was performed in the HDOCK server to study interactions with other proteins and ligands. In the docking analysis, docking scores of -254.7 and -269.55 were assigned for wildtype and mutants, with confidence scores of 0.891 and 0.9161, respectively, when docked with NOTCH2. It implies that the variant binds more strongly than the wildtype, as a greater negative docking score represents a more likely binding model [50]. NOTCH2, a member of the NOTCH family receptor, is associated with a distinctive oncogenic process [79]. It is frequently upregulated in several cancers, including hepatocellular carcinoma [80], gastric cancer [81, 82], glioblastoma [83], medulloblastoma [84], B cell malignancies [85], etc. This transmembrane receptor family contains extracellular epidermal growth factor-like (EGF) repeats domain, with several intracellular domains [86]. It has been reported that EGFR-BTB domain oligomerization activates downstream signaling cascade without EGF [87]. So, the better binding pose of the variant and NOTCH2 complex implies the overexpression of NOTCH2 signaling, followed by a greater risk for oncogenesis.

Two independent ligands (acetaminophen and Adderall) were also docked with native and variant models as the negative control. The results unveiled that the wildtype and mutant models don’t form non-specific interactions. For evaluating the change in dynamic characteristics of the protein owing to nsSNP, the molecular simulation was conducted for 100 nanoseconds using GROMACS software. The analysis showed that the wildtype structure possessed higher RMSD than the variant, and the same trend was observed for RMSF, radius of gyration, and SASA analysis. The nsSNP (rs10936599) alters the stability, compactness, flexibility, and solvent accessibility of the protein. According to RMSD, RMSF, radius of gyration, and SASA profile, the polymorphism seemed more stable than the wildtype.

Usually, nsSNPs modify the protein structure and function potentially [88]. Previous studies suggested that changes in protein stability are indeed connected to changes in function. It’s important to note that stability changes alone cannot reliably predict how a protein’s function will be affected [88]. Even though the overall structure of the variant seemed more stable, it might modify specific regions responsible for the protein’s function. Notedly, the non-coding SNP is situated near the Skp1/Btb/Poz Domain, which mediates protein-protein interactions. Hence, this variant potentially alters interaction with others.

Non-coding SNPs of MYNN were also studied because a mutation in non-coding regions can ultimately affect transcription, translation, and phenotype [89]. According to GWAS, about 90% of all SNPs associated with phenotypes are located in the non-coding region [90]. SNPs of 3 prime UTR regions and 5 prime UTR regions with introns were focused on as functional variants are mostly found in these regions [91]. The non-coding SNPs were subjected to RegulomeDB analysis to assess whether these variants disrupt the regulatory transcription factor binding sites [92]. This analysis exposed that most polymorphisms affected transcription factor binding or DNase peak, followed by motif hit and transcription factor binding + any motif + DNase peak. GTEx Portal was employed to explore genetic mutations, gene expression, and other molecular phenotypes in numerous reference tissues through eQTL, relative gene expression, and splicing quantitative trait loci [93]. Expression quantitative trait loci (eQTL) is a simple method for identifying potential candidate genes at risk sites [94]. The GTEx analysis demonstrated single tissue eQTL of SNPs in testes, with normalized expression represented in violin plots. Further, non-coding SNPs proceeded for analysis in PolymiRTS to distinguish SNPs that influence miRNA and their target locations [56], as these small, non-coding RNAs control gene expression post-transcriptionally [95]. Two polymorphisms were found: rs1920123 disrupting the conserved target site and rs75277808 generating a novel target site. Lastly, HaploReg v4.1 was utilized for annotating non-coding polymorphisms and forecasting their associations with diseases [55].

This study implicated that variant rs10936599 has a pathogenic role in the development of several diseases and cancers. It is also supported by GWAS Catalog [5] with the higher odd ratio for the G allele of rs10936599 and previously reported literature [8, 20]. However, this study needs further research and clinical evidence.

## Conclusions

Through a comprehensive bioinformatics approach, this study characterized rs10936599 of MYNN by unraveling its functional outcomes, structural modifications, molecular interactions, dynamics properties, and other properties. It also predicted a novel 3D structure of the complete protein sequence. This analysis can support further research in this field, ensuring a better understanding of this SNP and aiding in developing therapeutic treatments and drug discovery processes.

## Supporting information

S1 Fig Illustration of the secondary structures of A) native protein B) nsSNP. (TIF)

S2 Fig Schematic representation of the Ramachandran plots and ProSA-web Z-score plots. A) Ramachandran plot of wildtype MYNN structure. B) Ramachandram plot of rs10936599 structure. C) ProSA-web Z-score plot of wild structure. D) ProSA-web Z-score plot of variant structure.
(TIF)

S3 Fig Presentation of single tissue eQTL violin plots of non-coding SNPs. (TIF)

S1 Table List of gene enrichment terms of MYNN. (XLSX)

S2 Table List of Non-coding SNPs of MYNN. (XLSX)
