# Supplementary figures and images for: Comprehensive characterization of coding and non-coding single nucleotide polymorphisms of the Myoneurin (MYNN) gene using molecular dynamics simulation and docking approaches
Source: PLoS One. 2024 Jan 2;19(1):e0296361. doi: 10.1371/journal.pone.0296361 (PMC10760682; doi:10.1371/journal.pone.0296361)

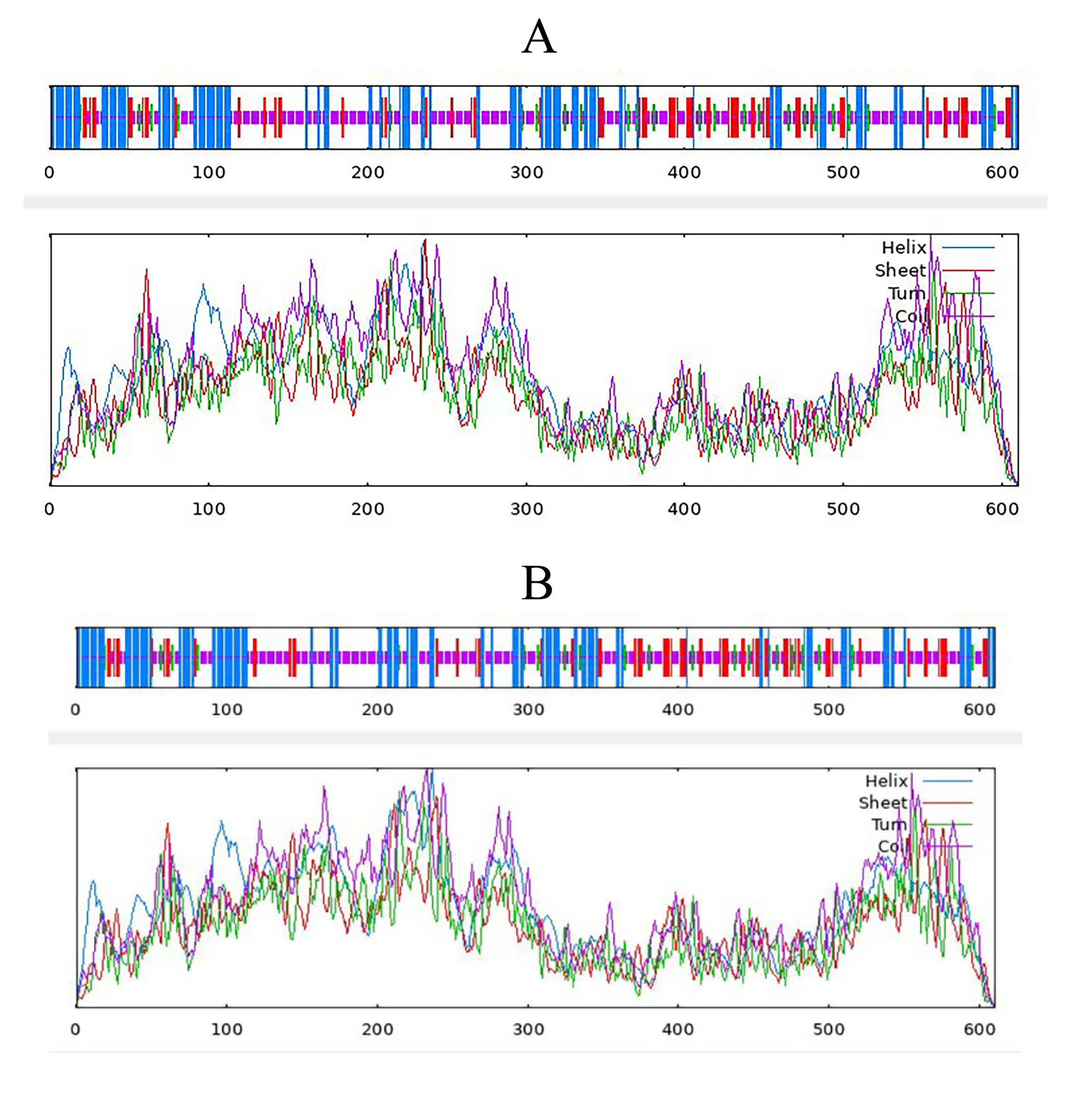

Supplement: S1 Fig — (TIF) [file pone.0296361.s001.tif]

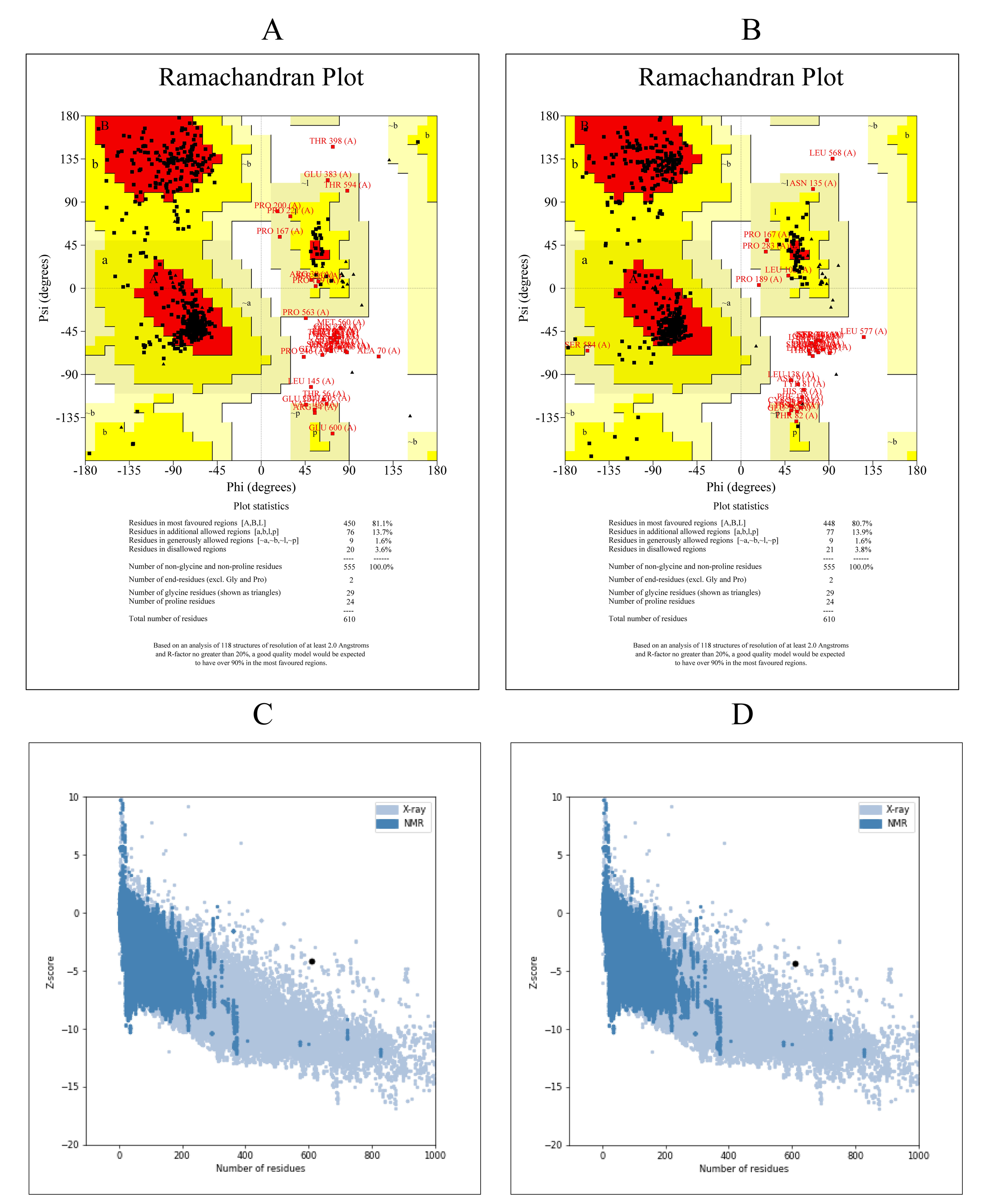

Supplement: S2 Fig — A) Ramachandran plot of wildtype MYNN structure. B) Ramachandram plot of rs10936599 structure. C) ProSA-web Z-score plot of wild structure. D) ProSA-web Z-score plot of variant structure. (TIF) [file pone.0296361.s002.tif]

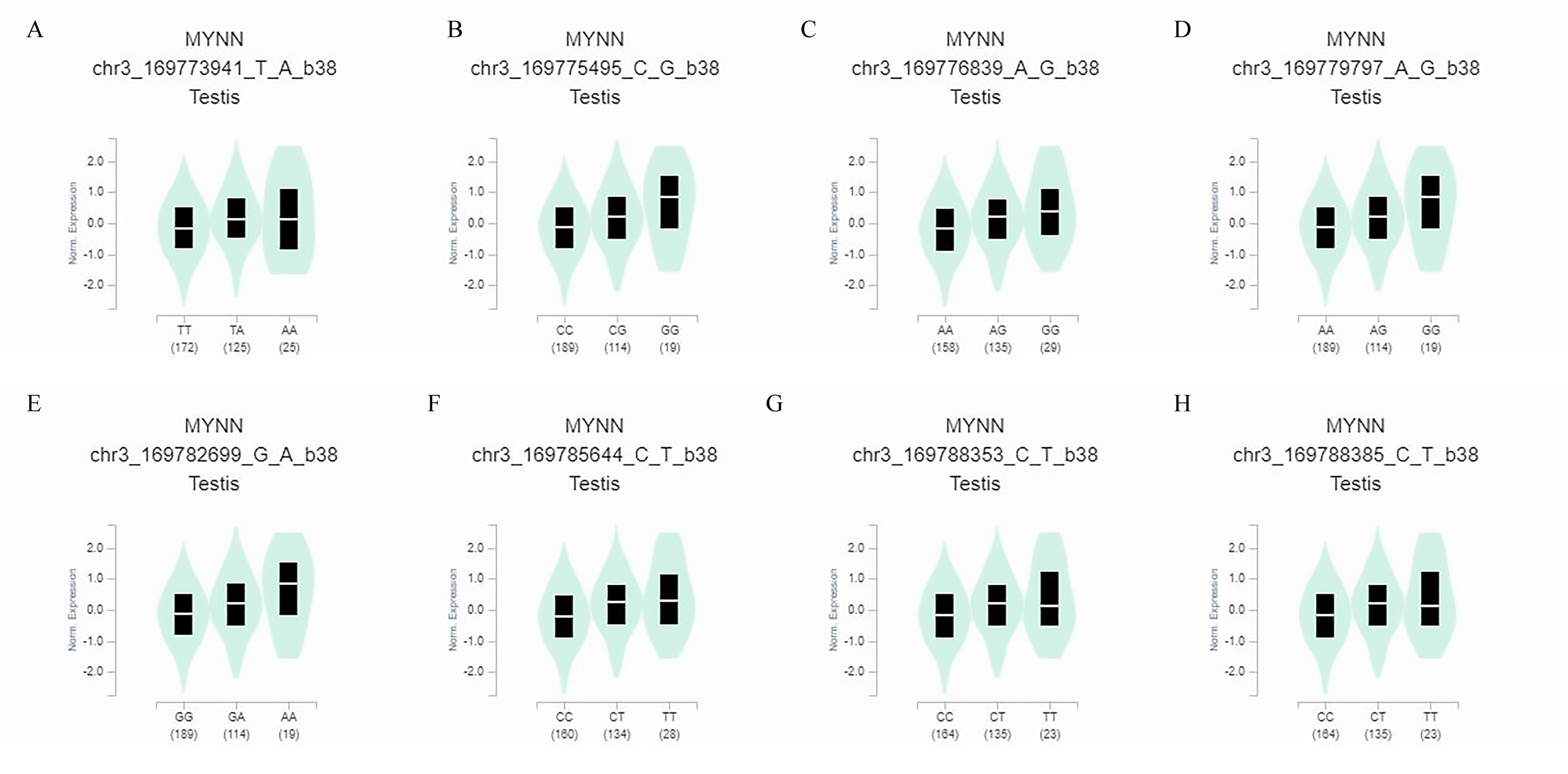

Supplement: S3 Fig — (TIF) [file pone.0296361.s003.tif]
